# Zebrafish xenograft as a tool for the study of colorectal cancer: a review

**DOI:** 10.1038/s41419-023-06291-0

**Published:** 2024-01-09

**Authors:** Camilla Maria Fontana, Hien Van Doan

**Affiliations:** 1 Department of Animal and Aquatic Sciences, Faculty of Agriculture, Chiang Mai University, Chiang Mai, Thailand; 2 Office of Research Administration, Chiang Mai University, Chiang Mai, Thailand

**Keywords:** Cancer models, Colorectal cancer

## Abstract

Colorectal cancer (CRC) is the second leading cause of cancer-related death, mostly due to metastatic disease and the fact that many patients already show signs of metastasis at the time of first diagnosis. Current CRC therapies negatively impact patients’ quality of life and have little to no effect on combating the tumor once the dissemination has started. *Danio rerio* (zebrafish) is a popular animal model utilized in cancer research. One of its main advantages is the ease of xenograft transplantation due to the fact that zebrafish larvae lack the adaptative immune system, guaranteeing the impossibility of rejection. In this review, we have presented the many works that choose zebrafish xenograft as a tool for the study of CRC, highlighting the methods used as well as the promising new therapeutic molecules that have been identified due to this animal model.

## Facts

1. CRC progression into the metastatic disease is one of the leading causes of cancer-related death; 80% of patients at the metastatic stage do not survive.
2. Zebrafish is an efficient animal model for studying human cancer and researching new therapeutics molecules.
3. The use of zebrafish xenografts to study CRC has opened the door for discoveries in the tumor–host interaction, as well as for the discovery of possible new treatments.

## Open questions

- Are zebrafish xenografts a suitable technique for the study of human cancer?
- How have zebrafish xenografts been used in literature for the study of CRC?
- Can zebrafish xenografts be used to develop and assess new therapeutic molecules against CRC?

## Colorectal cancer

**Fig. 1:**
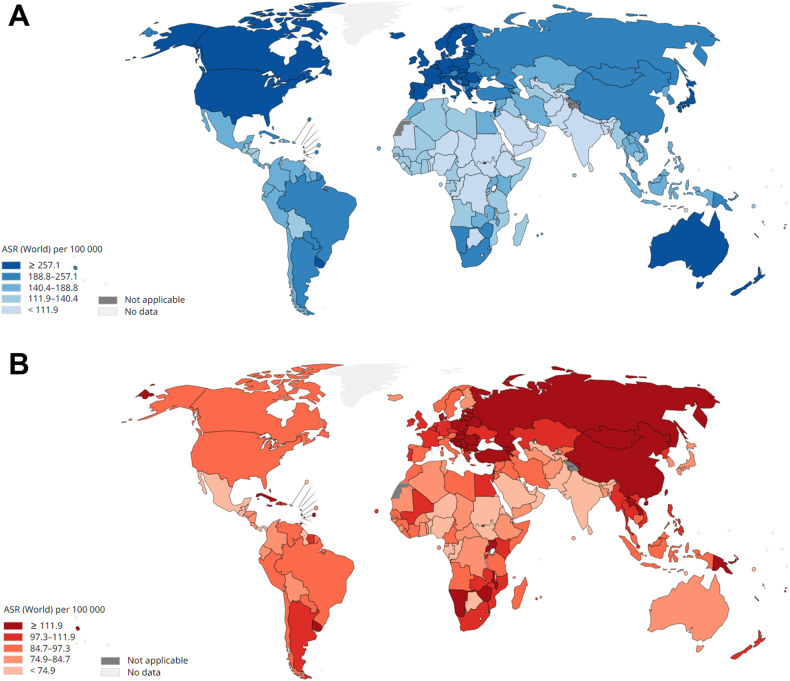
CRC incidence and mortality. **A** Estimated age-standardized incidence rates of CRC in 2020 globally, for all sexes and ages. **B** Estimated age-standardized mortality rates of CRC in 2020 globally, for all sexes and ages. Data source: GLOBOCAN 2020 Map production: IARC (http://gco.iarc.fr/today) World Health Organization, International Agency for Research on Cancer 2022. All rights reserved.

Colorectal cancer is the second most common cancer in women and the third most common in men, accounting for 10% of all annually diagnosed cancers and killing 930,000 people worldwide only in 2020, making it the second leading cause of cancer-related death [1, 2]. Usually regarded as one entity, CRC is in reality comprised of colon cancer and rectal cancer, which differ not only for their anatomic localization but also showcase different origins, metastatic patterns, and suggested treatments [3]. While the death rates vary geographically (Fig. 1) and among sexes, the incidence of CRC is only expected to increase, reaching 2–5 million new cases by 2035 [4]. This incredibly high death rate is due to metastatic disease [1]. In fact, more than 20% of United States CRC patients already show metastatic disease at the time of the first diagnosis, especially for adults older than 50 years of age [5]. While surgical resection of the primary tumor often guarantees prolonged survival, 80% of CRC patients at the metastatic stage will inevitably die [6].

**Fig. 2:**
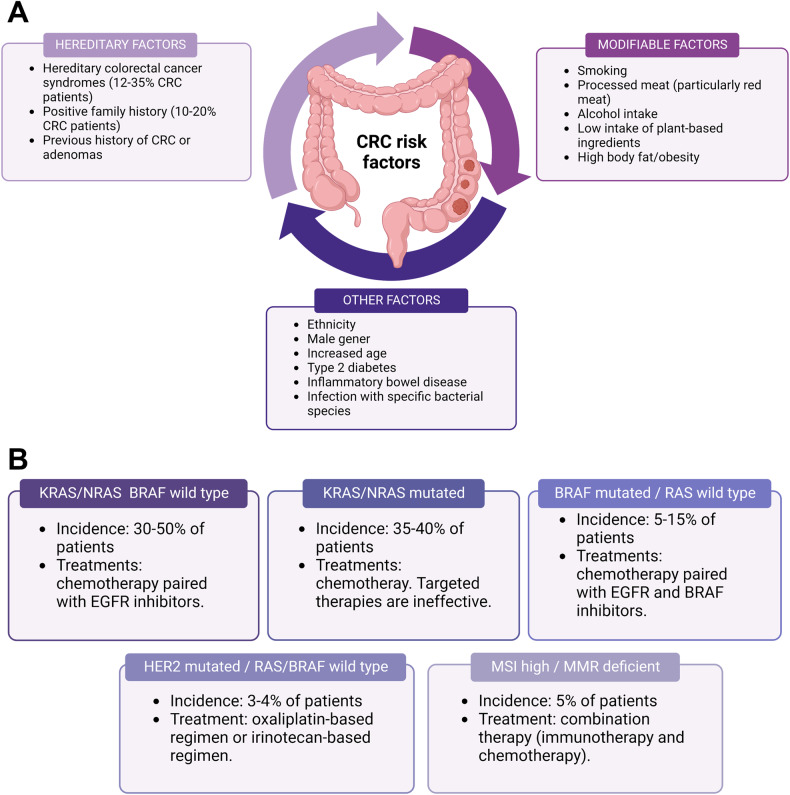
CRC risk factors and subtypes. **A** List of hereditary, modifiable and various other risk factors involved in colorectal cancer. **B** List of the five different subtypes of metastatic CRC with their incidence and treatment options following Sakata and collaborators [27]. Figure generated on Biorender.com.

There are several risk factors, both environmental and hereditary, that have been strongly correlated with CRC incidence (Fig. 2A). Many cancer susceptibility genes have been identified, usually with common single-nucleotide polymorphism associated with CRC risk; however, many of the aspects of CRC hereditability, which seems to account for 12–35% of the cases, are still up for debate [7–9]. Moreover, long-standing inflammatory bowel disease, type II diabetes, and previous history of CRC or adenomas also determine an increased risk for this cancer [10–13]. Several modifiable environmental factors can also increase the risk of CRC, such as smoking, excessive alcohol and red meat intake, obesity, and low intake of vegetables and fruits [14–17]. Other factors involved are gender and ethnicity, and even the infection with specific bacterial species such as *Fusobacterium nucleatum* and *Bacteroides fragilis* [4, 18, 19].

Normally, CRC arises when an aberrant crypt evolves into a polyp, also known as a neoplastic precursor lesion. This polyp will eventually progress into CRC, a process usually 10-to-15 years long. At the beginning of these processes, there is usually an accumulation of genetic and epigenetic alterations that cause the inactivation of tumor-suppressor genes and the activation of oncogenes [20, 21]. CRC usually progresses into invasion and metastasis, predominantly in the liver. The dissemination can start even when the primary carcinoma remains undetectable [22, 23]. Metastatic CRC is an extremely heterogeneous disease, and patients are divided into five distinct subtypes of CRC, based on the types of mutations they present (Fig. 2B).

Angiogenesis plays a key role in the progression of CRC, as blood vessels supply the tumor with nutrients and oxygen. In fact, CRC often presents aberrantly activated components of the VEGF pathways, resulting in increased lymphatic vessel density and permeability, which facilitates the dissemination of metastasis in the lymph nodes, as well as increased vascular permeability and vascular permeability angiogenesis [24].

Due to the increase in CRC screening programs, more early cancers can be identified. Some of these can be resected endoscopically more safely and cheaply. Surgery is the main curative treatment for non-metastatic CRC when endoscopy is not possible. Preoperative radiotherapy is also gaining popularity in reducing the risk of local recurrence, with chemoradiotherapy as the most frequent therapy. Local treatment is also a viable option for metastatic disease; for example, liver surgery is now considered low-risk, but this option might be less viable depending on the location and number of metastases. If local treatment is not possible, systemic therapy is usually chosen by tailoring it with patient-specific markers based on the disease subtype (Fig. 2B) [3, 24].

CRC therapies greatly impact the quality of life of CRC patients, often negatively. Frequent side effects of chemotherapy are cumulative neuropathy and liver toxicity, which add to the range of symptoms of the metastatic disease, requiring constant exercise, diet control, pain relief, and psychosocial support to improve the life of patients [4].

## Zebrafish as a xenograft model

*Danio rerio*, commonly known as zebrafish, is an animal model first used in research in the early 1980s for the study of developmental biology and utilized for cancer research as early as 1982. Many aspects led to consider zebrafish one of the most popular animal models in research, namely: high fecundity and external fertilization, fast development (at 72 h post-fertilization, all core vertebrates features are already developed), and optical clarity during the larval stage. All these characteristics set it apart from mammalian models, which require a longer time and are unsuitable for high-throughput screening [25, 26]. Moreover, zebrafish also present a 70% similarity with the human genome, with the conservation of even the epigenetic marks [26, 27].

**Fig. 3:**
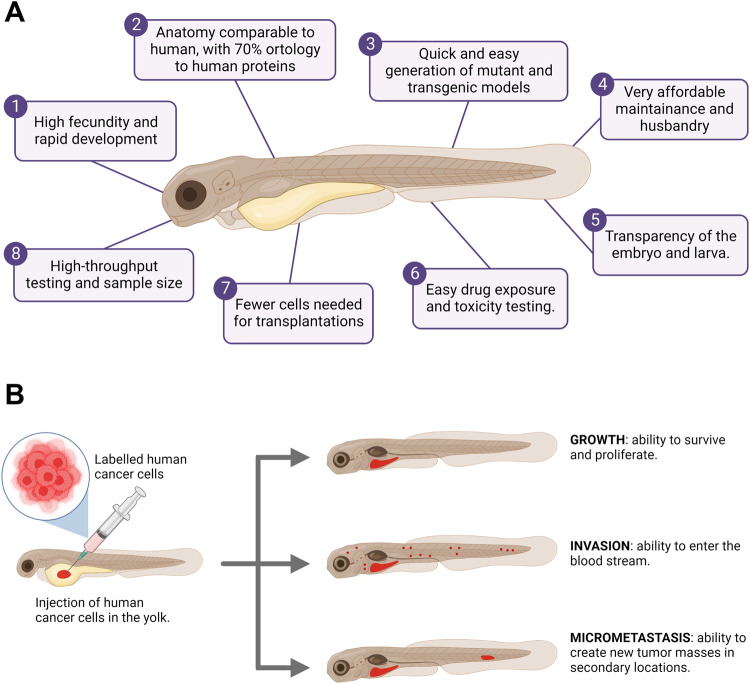
Zebrafish as a xenograft model. **A** Summary of the advantages of Danio rerio as an animal model in research. **B** Schematization of the signs of cancer progression in zebrafish xenografts, starting with the injection of fluorescent human cancer cells in the yolk of a 48 hpf zebrafish (left). Panel modified from Gamble and collaborators [25]. Figure generated on Biorender.com.

With the current technology, the generation of transgenic and mutant models is relatively simple, and these models are precious tools for the study of many human malignancies, such as cancer, as well as drug discovery and toxicity evaluation [28–32]. While it would be wrong to assume that drug effects can be consistently compared between zebrafish and humans, evidence suggests that existing drugs often work in this animal model and that zebrafish can be used to identify novel bioactive molecules [33]. The many advantages of the zebrafish animal model can be summarized in Fig. 3A.

One of the strongest advantages of using *Danio rerio* for cancer research lies in the technique known as xenograft transplantation, which is the relocation of living cells from one species to another. Specific to the objective of studying cancer, it is the relocation of living human tumor cells into a zebrafish embryo or adult [25]. While this technique is possible in many other animal models, the zebrafish embryo and larva specifically offer many advantages. During the first 30 dpf (days post fertilization), zebrafish only possess innate immune cells, completely lacking an adaptative immune system, guaranteeing the impossibility of rejection [34]. Moreover, zebrafish larvae are tiny and can be kept in Petri dishes or 96-well plates for easy handling and maintenance. Furthermore, they can survive at temperatures up to 36 °C, which is very close to human cell culture conditions, despite their preferred environmental temperature being 28 °C [34–36]. Finally, the xenotransplantation in one zebrafish embryo requires a much smaller number of cancer cells if compared to mammalian xenograft models, which is especially important for patient-derived cancer cells, which are available in a small finite number [36, 37].

Many types of cancer cell lines have already been proven to be able to proliferate in the zebrafish yolk, as well as invade the rest of the organism forming metastasis (Fig. 3B), namely: neuroblastoma [38], melanoma [39], leukemia [40], prostate [41], ovarian [42], colorectal cancer [43], among many others.

Cancer metastasis is one of the biggest threats to a patient’s survival, and thus it is fundamental to establish metastatic animal models, to gain a better understanding of the cell migration mechanisms and ability of various human tumors [44]. Zebrafish allows in vivo imaging to monitor the interaction between the cancer cells implanted and the animal system, opening many possibilities for the study of invasion and dissemination. In addition, angiographic zebrafish models such as the Tg(*fli*:eGFP) zebrafish line, a transgenic model with GFP-labeled blood vessels, allow for the in vivo study of the interaction between endothelial and cancer cells [44–46] and the use of confocal microscopy opens up many possibilities for the study of single-cell behavior of cancer cells in a complex system [44].

Despite rodent models being the standard for drug toxicity testing, even in the context of cancer, embryonic and larval zebrafish are precious tools that allow for quick assessment of systemic toxicity by testing multiple concentrations of many small molecules [47] while only requiring micro-liter volumes of the media, thus only using minimal amounts of chemicals. Furthermore, the entirety of the zebrafish body can be captured to analyze any phenotypic effect, collecting data in a simple and fast manner from a high number of samples [47–49].

While zebrafish has proven itself to be an up-and-coming promising and useful model for the studying of human cancer and new possible therapeutic approaches, it is important to state that this model does not come without its limitations. The most commonly used approach of drug dosing via immersion in water presents some challenges, such as compounds with low water solubility, and this approach also exposes the whole fish body to the compound, a methodology that differs greatly from mammalian drug intake. Moreover, questions still remain regarding the internal dose received by a fish exposed to a compound via water immersion [44]. Finally, it is important to note that while zebrafish shares 70% of homology in disease-related genes with humans, there are still many differences in gene functions that need to be highlighted, and it is important to note that zebrafish also have an increased ability to regenerate multiple tissues, compared to mammals [50]. These characteristics must be fully noted when performing cancer studies using zebrafish as an animal model.

## Colorectal cancer cell lines used for zebrafish xenografts

**Fig. 4:**
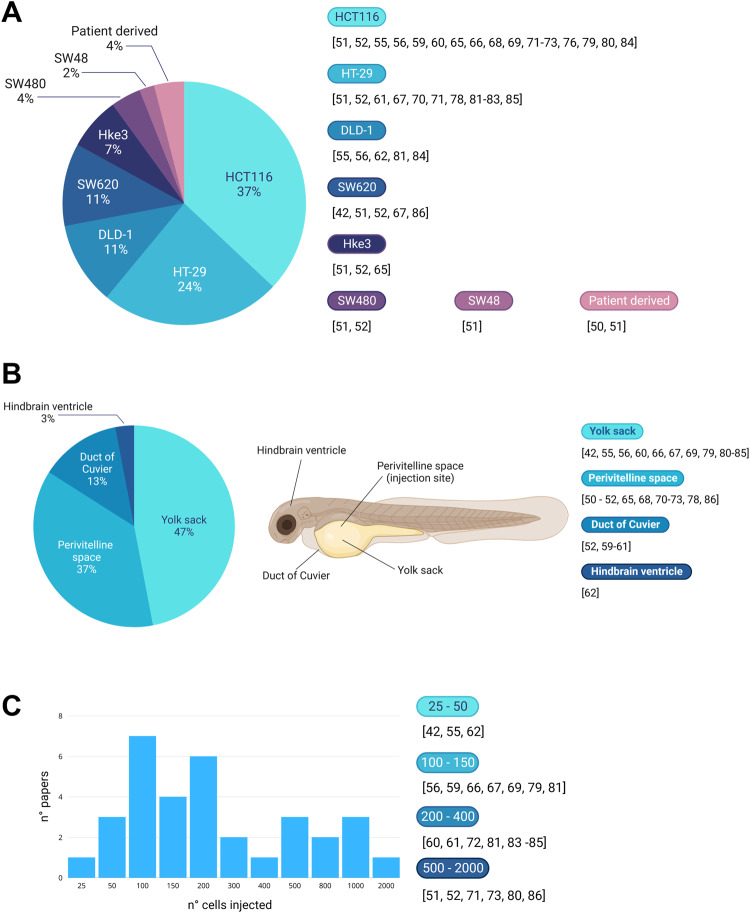
CRC cell lines specifics for zebrafish xenograft. **A** Schematization of the CRC cell lines used in the context of zebrafish xenografts. On the left: the proportions of each line used are represented as percentages. On the right: a bibliography of each study that utilizes each cell line (or patient-derived cells). **B** Schematization of the injection sites used in zebrafish xenografts. The pie chart shows the proportion of injection sites used in literature, expressed as percentages. The localization of the injection sites is shown on the right. **C** The column chart on the left summarizes the number of studies that use a specific number of injected CRC cells, between 25 and 2000. On the right are listed the studies, grouped by the number of cells used. The numbers in the graph do not correspond to the number of papers listed, as several papers make use of different cell numbers for their experiments. Figure generated on Biorender.com.

Several existing studies have documented the use of colorectal cancer cells, patient-derived or not, in zebrafish xenograft models. However, as many of these tumor lines exist and are readily available, it is important to choose the one more suitable for each research. While studies with a patient-derived approach are currently extremely infrequent [50, 51], the vast majority of research has been performed on only a few CRC lines. The most commonly used, as can be observed in Fig. 4A, is the HCT116 human CRC line, being chosen 37% of the time. This cell line is followed by the HT-29 human CRC line, with a frequency of 24%, and by DLD-1 and SW620, both being used 11% of the time.

Notably, different cell lines present highly different engraftment rates, with engraftment being defined as the frequency of xenografts that present a tumor consisting of at least 30 tumor cells [52]. For example, in a paper from Póvoa and collaborators, based on their engraftment rates, the SW480 and SW48 lines can be considered regressors, as they engraft poorly with a rate of only 20–30%, while HT-29, SW620, HCT116, and Hke3 are progressors, with an engraftment rate was higher than 80% [52]. Fior and colleagues confirmed the poor engraftment rate of the SW480 cell line and found the cell lines SW620, HCT116, Hke3, and HT-29 all with an engraftment rate higher than 70% [53]. CRC cell lines also differ in their proliferation rates, which appear to be greatest for the SW620 and HCT116 lines, and lowest for the Hke3 line [53].

As angiogenesis is an essential aspect in the study of the tumor–host interaction, it is important to be aware that while SW480, SW620, HCT116, and Hke3 show a well-vascularized periphery with large vessels that usually do not infiltrate the tumors, HT-29 forms highly vascularized tumors with a dense network of infiltrated vessels [53]. This discrepancy can easily be explained by the higher expression levels of VEGF in this human CRC line [53, 54].

The ability of cells to form metastasis is another essential hallmark of cancer, and it is also another aspect in which the potential of each CRC cell line can differ significantly. In fact, the Hke3 line shows the lowest metastatic potential, while SW480 and HT-29 show the highest, and SW620 and HCT116 are in an intermediate position [53]. DLD-1 also appears to have a lower metastatic potential when compared to the HCT116 line [55]. As the expression level of Fascin1 is strongly involved in tumor invasion and metastasis, it is also essential to state that HCT116 is the CRC cell line with the highest level of Fascin1 expression, while DLD-1 is the line with the lowest Fascin1 expression level [56].

These many differences should be taken into consideration when choosing the ideal cell line for any specific aim and experiment.

## Zebrafish xenograft injection sites and cell number

Zebrafish is a very versatile tool that offers several different locations for the engraftment of tumor cells. In literature, four locations have been used for the engraftment of CRC cells: the yolk sack, the perivitelline space (PVS), the duct of Cuvier, and the hindbrain ventricle.

The yolk sack is the site established by the standard protocol because it is an acellular compartment that constitutes a delimited and accessible space where cells can be easily visualized. It is commonly used for the study of survival, division, proliferation, and migration, but it offers a microenvironment that might not be ideal for the study of human tumors [56, 57]. In fact, the yolk is a viscous syncytium that provides an environment close to a cell suspension stage, which is far from ideal for anchorage-dependent growth [58]. Nevertheless, as can be observed in Fig. 4B, the yolk sack is the preferred injection site for almost half of the totality of papers that have studied CRC using a zebrafish xenograft approach, and this is probably due to the ease of injection. Hopefully, more studies in the future will choose the PVS injection approach.

The PVS is located distally between the periderm and the yolk syncytial layer. It is an avascular region ideal for identifying newly formed vessels and studying migration and metastatic behavior [57]. While it is considered the ideal injection site in zebrafish, it is technically more difficult to successfully inject tumor cells in the PVS, compared to the yolk sack [56, 57]. Despite the technical difficulties, the number of studies that make use of this injection site has greatly increased in recent years, and to this day, 37% of all CRC research with zebrafish xenografts showcase tumor injection in the PVS (Fig. 4B).

The Duct of Cuvier, or common cardinal vein, is the injection site that allows the introduction of tumor cells directly into the bloodstream, highlighting it as the perfect technique to study migration, invasion, and metastatic potential [57]. However, only a few papers have injected CRC cells in the Duct of Cuvier, with very interesting results concerning migration and metastasis [52, 58–60].

Finally, a single paper has identified the hindbrain ventricle as an injection site for the study of the metastatic behavior of human CRC cell lines, with promising results [61].

The number of tumor cells injected is one of the many parameters that need to be determined when performing xenograft experiments. In literature, the range between 25 and 2000 cells has been used for zebrafish when injecting CRC cells, and the most popular cell number appears to be between 100 and 200 cells (Fig. 4C). However, there seems to be no correlation between the number of cells injected and the site of injection used as, for example, the injection in the Duct of Cuvier has been performed with a number of cells between 150 and 1000 [52, 58–60], and the same variety in cell numbers can be observed for the yolk sack and the PVS (Fig. 4C).

Interestingly, a single paper focused on patient-derived xenograft performed its experiments by successfully implanting a tumor fragment of unspecified dimensions in the PVS [51].

## Transgenic zebrafish lines

One of the main advantages of using zebrafish as an animal model is the availability of many transgenic lines that aid the visualization of structures and pathways as they change and develop in the embryo and larvae. Some of these reporter lines can be extremely useful for studying the interaction between a tumor and its host. Interestingly, less than half of the papers revolving around the study of CRC using a zebrafish xenograft approach make use of these incredible tools, but the results are surely notable.

As angiogenesis is a significant factor that requires study regarding tumor progression, it is no surprise to find an abundance of papers that utilize transgenic lines aimed at the in vivo visualization of blood vessels. First developed by Lawson and Weinstein in 2002, the Tg(f*li1*:eGFP) transgenic line is now the most established tool for observing blood vessels in zebrafish. Fli1 is a known endothelial cell marker expressed throughout zebrafish development. The eGFP in this transgenic line can be detected in the lateral mesoderm already during early somitogenesis, and its expression persists in all blood vessels, hematopoietic cell types, and the jaw mesenchyme [62]. An alternative to this transgenic line is the Tg(*kdrl*:eGFP) transgenic line that, instead of fli1, uses the promoter for the vascular endothelial growth factor receptor 2 gene (VEGFR2, also known as KDR) [63]. The two lines provide very similar outcomes when it comes to blood vessel visualization, but while several researchers have chosen the Tg(f*li1*:eGFP) line for their study of CRC in zebrafish [52, 59, 60, 63–70] only one group has chosen the Tg(*kdrl*:eGFP) instead [71].

In the interest of studying the host’s immune response in the presence of a tumor, transgenic lines highlighting macrophages and neutrophils have also been utilized for the study of CRC in engraftment models, even if on much rarer occasions. Macrophage transgenic lines revolve around *mpeg1* (macrophages expressed gene 1) and showcase macrophage-specific fluorescence. While the first of such lines was generated in 2011 [72], variations associated with different fluorescent proteins have been generated. Specifically, for the study of CRC, Maradonna and collaborators have chosen the Tg(*mpeg1*:eGFP) transgenic line [60], while Póvoa and colleagues have selected the Tg(*mpeg1*:mCherry-F) transgenic line and the Tg(*mpeg1*:mCherry-F; *tnfa*:eGFP-F), which also showcases TNFa positive cells in green fluorescence [52].

Neutrophil transgenic lines were used as their target for the neutrophil-restricted granule protein myeloperoxidase Mpo, also known as Mpx. The first of these transgenic lines was generated in 2006 [73], and to studying the interaction of CRC with the zebrafish immune system, as of now, the Tg(*mpx*:GFP) line has been used on various occasions [51, 64, 67].

## Tumor size and growth analysis

One of the main interests when performing a zebrafish xenograft experiment is the analysis of the tumor size, as well as its growth or growth inhibition. The zebrafish’s optical properties make these experiments extremely easy, as tumor cells marked with a fluorescent dye before engraftment can easily be visualized in vivo, even with a simple stereo-fluorescent microscope.

**Fig. 5:**
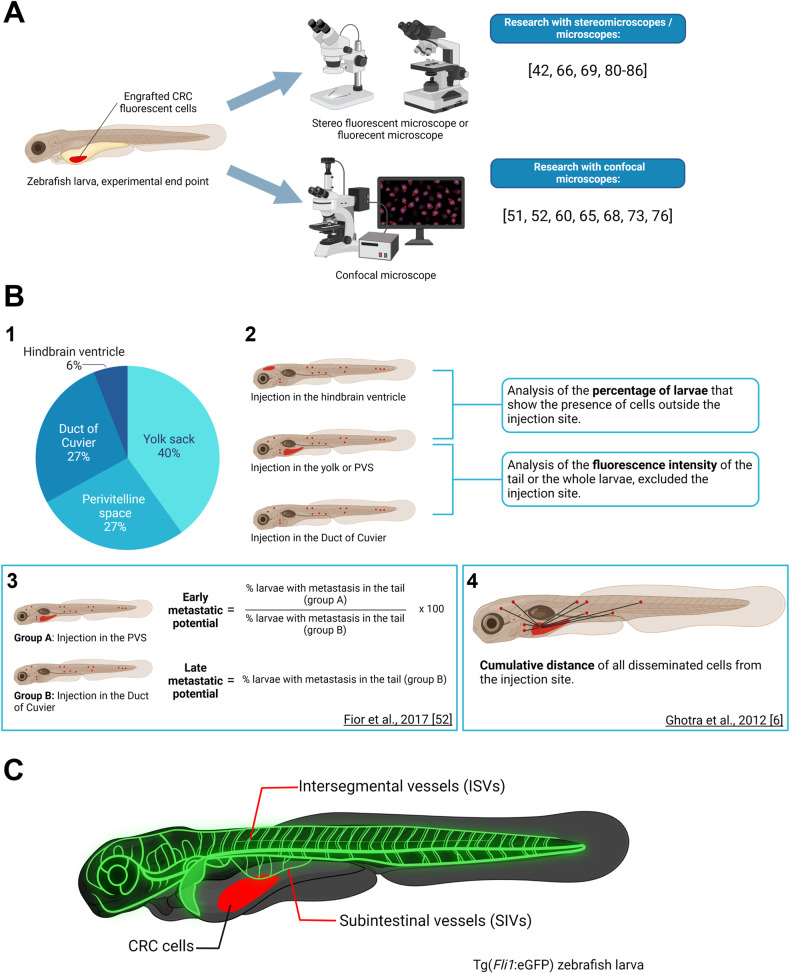
CRC xenografts visualization. **A** Summarization of the two main types of imagining tools utilized for the analysis of the tumor size or tumor growth. On the right is the bibliography of studies presenting one or the other approach. **B1** Pie chart portraying the proportion of papers presenting zebrafish xenografts with the various injection sites: yolk sack, perivitelline space (PVS), duct of Cuvier, and hindbrain ventricle. **B2** Schematization of the two main types of analysis of metastatic potential: analysis of the percentage of larvae that present metastasis, or analysis of the fluorescence intensity of the metastasis. **B3** Metastatic potential method used by Fior and collaborators [53]. **B4** Tumor dissemination capacity method used by Ghotra and colleagues [66]. **C** Schematization of the blood vessels’ structure in a Tg(*Fli1*:eGFP) zebrafish larva. The CRC cells engrafted in the larva are highlighted in red. The intersegmental vessels (ISVs) and the subintestinal vessels (SIVs) are highlighted in glowing green. Figure generated on Biorender.com.
- Use of confocal microscope. Analysis of tumor size via cell counter of Z stacks of 5 µm using the formula “tumor size = AVG (Zfirst, Zmiddle, Zlast)×total number of slices/1.5” [52, 63, 66] or the formula “tumor size =(Zfirst + Zmiddle + Zlast)/total number of slices × 1.5” [51, 74]. The 1.5 correction is due to the fact that CRC cells present a diameter of 10–12 µm [52] (Fig. 5A).

The methods surrounding the analysis of the tumor size in CRC-focused experiments in literature are split into two different techniques:

- Use of stereo fluorescent microscope, fluorescent microscope, or inverted fluorescent microscope for image acquisition. Analysis of tumor size as the integrated density of the fluorescent intensity (Fig. 5A).

Exceptions to these two methods are the works of Maradonna and colleagues and Roel and colleagues, which use a confocal microscope and calculate the tumor size as the simple sum of the integrated density of the fluorescent intensity of each Z stack [59, 73].

While using a confocal microscope offers a higher level of precision and image definition, using a lower microscope is more time-efficient and is usually preferred for bigger sample sizes.

## Invasion, dissemination and metastasis analysis

Due to the high mortality of CRC metastatic disease [5], the study of metastasis and possible therapeutic solutions to stop the metastatic progression in zebrafish xenograft CRC models is imperative. To date, several papers have provided different approaches to studying the metastatic potential of CRC cell lines and the effect of various therapeutic molecules on the cells’ ability to disseminate and proliferate outside of the injection site (Fig. 5B). In the case of injection in the hindbrain ventricle, yolk sack, or PVS, the most frequently used measure of the metastatic potential seems to be the percentage of larvae exhibiting tumor cells outside of the injection site [5, 55, 59, 64, 69, 75, 76]. An individual with metastasis is often described as having at least one [61] or three [55, 75] tumor cells outside of the injection site. In the case of injection again in the yolk sack or PVS, or the Duct of Cuvier, another prevalent method to determine the metastatic potential is the analysis of the fluorescence intensity of the disseminating cells, either only focused in the tail region [5, 58, 60] or in the whole animal [59, 70] (Fig. B2).

Fior and collaborators have proposed a fascinating approach that separates the early metastatic potential from the late metastatic potential (Fig. B3) since the metastatic efficiency could vary depending on whether a tumor cell can detach from the primary tumor or is directly injected into the bloodstream [53]. Therefore, this approach should be taken into consideration for in-depth analyses of the metastatic potential and makes use of both injections in the PVS and the Duct of Cuvier.

Another interesting approach has been presented by Ghotra and colleagues (Fig. B4), which takes into consideration the tumor dissemination capacity, measured as the cumulative distance traveled by disseminating cells starting from the yolk sack as the selected injection site [66].

Another alternative to measuring the total fluorescent intensity of disseminated cells is the count of the disseminating cells or micrometastasis areas [64, 77].

Having taken a vision of all these different methods, it is important to remember that the dissemination process of a human xenograft in zebrafish might not recapitulate exactly how this process appears in humans, and the visualization of the disseminated cells themselves could be subjected to bias due to the lack of dyes specific for the nuclei in the works here presented. Because of this, we advise proceeding with caution when discussing and studying metastasis in zebrafish xenograft models.

## Angiogenesis analysis

Even though CRCs are highly vascularized tumors and increased angiogenesis is associated with poor prognosis and tumor relapse [24], only a few papers use zebrafish xenograft models to study the interaction between CRC and the blood vessels of the host. Despite the scarcity of literature, the special optical qualities of zebrafish larvae and the availability of transgenic lines that allow for the in vivo visualization of blood vessels, such as Tg(f*li1*:eGFP) and Tg(*kdrl*:eGFP), have enabled very interesting results.

Two are the different analysis strategies that have been used to determine the angiogenic activity in CRC zebrafish xenograft models:

- Analysis of the inhibition of intersegmental vessels (ISVs) and subintestinal vessels (SIVs) growth as a measure of their length (Fig. 5C), as well as the rough percentage of embryos/larvae that express a clear angiogenic phenotype as the percentage of embryos presenting ectopic vessels [64, 67].
- Analysis of the total vessel density (TVD) by using the maximum z-projection and the formula “TVS = GFP area/tumor area” [52, 59, 74] and analysis of the vessel infiltration (VI) by using the maximum z-projection and the formula “VI = GFP area/central tumor area” [52, 74].

Both approaches have proven useful, and one should be used over the other based on available tools.

## Antibodies for immunofluorescence

Due to the difficulties of having samples with cells of two different organisms (human and zebrafish), only a few projects have attempted to perform immunofluorescence on CRC zebrafish xenograft models. The most popular target for immunofluorescence is Caspase 3, a well-known marker of cell death through apoptosis. Anti-Caspase3 antibodies have been utilized to assess the level of apoptosis induced by various therapeutic molecules against CRC [52, 63, 66, 74]. The assessment of the mitotic activity in the tumor has been performed with two different strategies: while some researchers have chosen to stain the tumor with DAPI [52, 66] simply, Costa and collaborators have chosen to use an antibody anti-phospho histone H3 instead [64]. Another handy tool in immunofluorescence for zebrafish xenograft is the anti-Ki67 antibody, which allows the visualization of proliferating cells while guaranteeing no cross-reaction with the zebrafish host, as Ki67 is an exclusively human marker [52, 63]. Antibody against Mucin 2 has also been used on one occasion on CRC zebrafish xenografts. Mucin 2 expression has been correlated with proliferation in CRC; thus, it is a parameter worth analyzing [69]. Finally, considering the importance of utilizing transgenic fluorescent lines for the study of the interaction between the tumor and the host, antibodies against GFP are a handy tool in the immunofluorescence of xenograft samples and should be kept in high consideration [71]. All antibodies mentioned can be found in Table 1.

**Table 1:** List of all antibodies used on CRC zebrafish xenografts in literature.

Target	Function	AB used	Concentration	Publications
Caspase 3	Marker of apoptosis	Anti-Activated Caspase3 rabbit, Cell signaling code #9661	1:100	[63, 74]
		Anti-Activated Caspase3 rabbit, CST	Unknown	[52]
			1:100	[66]
Ki-67	Marker of proliferation	Anti-Ki67 mouse, Leica-Novo-castra, cat#NCL-Ki67-MM1	1:100	[63]
			Unknown	[52]
Phospho Histone H3	Marker of mitotic activity	Anti-phospho Histone H3 rabbit, Merck Millipore, cat#06-570	1:1000	[63]
Mucin-2	Marker of cell differentiation	Anti-Mucin-2 SC-23171	Unknown	[68]
GFP	Green Fluorescent Protein	Anti-GFP mouse, Roche #11814460001	1:100	[74]

## Therapeutic molecules against CRC tested in zebrafish xenograft models

The zebrafish xenograft model has been used to assess the efficacy of a great variety of compounds and therapeutic molecules (Table 2). It has been utilized to validate the effect of several chemotherapy regimens already commonly used against CRC in human patients, as well as FDA-approved drugs, proving that the results in the animal model can recapitulate the results in the patients [50, 52, 75]. Zebrafish have also been used to validate the effects of novel compounds, with promising results that open doors to developing new drugs against CRC [56, 65, 78–81]. Finally, CRC zebrafish xenograft has been used to validate the effect of several natural compounds, such as bromelain extracted from pineapple [82], polysaccharides derived from the orchid *D. officinale* [83], a lectin from the mushroom *L. sulphureus* [68], Deflamin from the legumes *L. albus* [71], compounds derived from marine sponges [84] as well as compounds of bacterial origin [85].

**Table 2:** List of therapeutic molecules tested against CRC in zebrafish xenograft models and their effect on the tumor cells.

Molecule	Effect	Publication
1,2,4-triazine derivative MM-129	Anti-tumor activity	[78]
Anandamide	Anti-tumor, anti-angiogenic and antimetastatic activity	[59]
Bromelain	Anti-tumor activity	[81]
Chemotherapy regimens (5-FU, FOLFIRI, FOLFOX, FOLFOXIRI)	Anti-tumor activity	[50, 52]
*Dendrobium officinale* polysaccharides	Anti-tumor activity	[82]
Dibenzyl tetrasulfide	Anti-tumor activity	[80]
*Exiguobacterium acetylicum* cyclic dipeptides	Anti-tumor, mitochondria-mediated apoptotic activity	[79]
Fascin1 inhibitor G2	Anti-tumor and antimetastatic activity	[55]
IMP2 inhibitors	Anti-tumor activity	[86]
*Klebsiella pneumonia*e microcin E492	Anti-tumor activity	[86]
*Laetiporus sulphureus* Lectin	Anti-tumor and anti-angiogenic activity	[67]
*Lupinus albus* Deflamin	Anti-tumor and anti-angiogenic activity	[74]
N-Heterocyclic carbene iron complexes	Antiproliferative activity	[58]
Raltegravir	Anti-tumor and antimetastatic activity	[75]
rhenium(I) tricarbonyl-based complexes	Anti-tumor, anti-angiogenic and antimetastatic activity	[64]
Sponge-derived crambescidine-816	Anti-tumor activity	[73]

## Limits of the zebrafish xenograft model

Despite all the advantages of using zebrafish to study human cancer, it is important to also consider the drawbacks of this model. First and foremost, an added complexity in this animal model is the fact that zebrafish often have more than one orthologue for many human genes, due to a teleost-specific whole genome duplication. Moreover, even in those cases where a human gene corresponds to only one zebrafish gene, the latter rarely produces a highly conserved protein. The consequence is that it is difficult to assess which proportion of drugs will reliably target the same protein in humans and zebrafish [33]. It is also important to note that there are not many published comparisons on the cancer behavior in different injection sites, zebrafish stages or incubation conditions, and the interpretation of these studies is often more complex due to the lack of control experiments with non-cancer xenografts or different cancer cell lines [33].

Finally, on a more technical note, it is important to note that most studies are developed by using membrane and cytoplasmic dyes to color the tumor cells. These dyes are unable to properly discriminate the dead cells from the alive ones, resulting in a biased over-estimation of tumor masses and growth [58]. In addition to this, while the ideal temperature for zebrafish is 28 °C, the best temperature for human tumor proliferation is 37 °C, a 9 °C increase. To perform these experiments, zebrafish are usually kept between 34 and 36 °C, which is not an ideal condition, but it does not increase zebrafish mortality [35].

## Future perspectives

While zebrafish has very clearly established itself as an excellent model for the study of human CRC, it is important to highlight that most of the studies these days revolve around the use of stable cancer cell lines maintained in a laboratory environment. Indeed, these cell lines are dramatically different from patient tumor cells as they are severely lacking heterogeneity. The expansion of the use of patient-derived tumor cells will not only limit this issue but will also improve the accuracy of tumor drug-response studies. Zebrafish will surely have an impact on personalized medicine in the near future, as it will be incorporated into the clinical setting due to its advantages compared to rival models [57] and this combinatorial approach will likely play an important role in the evolution of cancer therapy.

## Conclusions

The Zebrafish xenograft is an exceptional tool for studying human cancer in a more cost-effective and less time-consuming way while also guaranteeing a sample size that is not obtainable with mammals. Colorectal cancer is the second-leading cause of cancer-related deaths worldwide, especially due to metastatic disease, and as such, it is imperative that we understand more about this cancer, as well as find new therapeutic molecules that can drastically change the prognosis. Zebrafish have been proven to be a vital tool for this aim, and the many studies here summarized have shown how zebrafish xenografts can easily allow for the discovery of pathways involved in the CRC progression and metastasis, as well as new possible therapeutic molecules against it. This review will offer all the information needed to fellow researchers who are interested in using this exceptional tool for the study of CRC.
